# Supplementary material for: Resolving the full spectrum of human genome variation using Linked-Reads
Source: Genome Res. 2019 Apr;29(4):635–45. doi: 10.1101/gr.234443.118 (PMC6442396; doi:10.1101/gr.234443.118)
Supplement: Supplemental Material [file supp_gr.234443.118_Supplemental_longranger-2.2.zip › longranger-2.2.tar.bz2!/lr2.2-public/lib/stan/cmdstan/stan/doc/api/html/classstan_1_1optimization_1_1_b_f_g_s_line_search-members.html]

Stan: Member List


|  |  |
| --- | --- |
|  | Stan  2.5.0  probability, sampling & optimization |


- Main Page
- Related Pages
- Namespaces
- Classes
- Files

- Class List
- Class Index
- Class Hierarchy
- Class Members

All Classes Namespaces Files Functions Variables Typedefs Enumerations Enumerator Friends Macros Pages

- stan
- optimization
- BFGSLineSearch

stan::optimization::BFGSLineSearch< M, QNUpdateType, Scalar, DimAtCompile > Member List

This is the complete list of members for stan::optimization::BFGSLineSearch< M, QNUpdateType, Scalar, DimAtCompile >, including all inherited members.

|  |  |  |
| --- | --- | --- |
| \_alpha | stan::optimization::BFGSMinimizer< ModelAdaptor< M >, QNUpdateType, Scalar, DimAtCompile > | protected |
| \_alpha0 | stan::optimization::BFGSMinimizer< ModelAdaptor< M >, QNUpdateType, Scalar, DimAtCompile > | protected |
| \_alphak\_1 | stan::optimization::BFGSMinimizer< ModelAdaptor< M >, QNUpdateType, Scalar, DimAtCompile > | protected |
| \_conv\_opts | stan::optimization::BFGSMinimizer< ModelAdaptor< M >, QNUpdateType, Scalar, DimAtCompile > |  |
| \_fk | stan::optimization::BFGSMinimizer< ModelAdaptor< M >, QNUpdateType, Scalar, DimAtCompile > | protected |
| \_fk\_1 | stan::optimization::BFGSMinimizer< ModelAdaptor< M >, QNUpdateType, Scalar, DimAtCompile > | protected |
| \_func | stan::optimization::BFGSMinimizer< ModelAdaptor< M >, QNUpdateType, Scalar, DimAtCompile > | protected |
| \_gk | stan::optimization::BFGSMinimizer< ModelAdaptor< M >, QNUpdateType, Scalar, DimAtCompile > | protected |
| \_gk\_1 | stan::optimization::BFGSMinimizer< ModelAdaptor< M >, QNUpdateType, Scalar, DimAtCompile > | protected |
| \_itNum | stan::optimization::BFGSMinimizer< ModelAdaptor< M >, QNUpdateType, Scalar, DimAtCompile > | protected |
| \_ls\_opts | stan::optimization::BFGSMinimizer< ModelAdaptor< M >, QNUpdateType, Scalar, DimAtCompile > |  |
| \_note | stan::optimization::BFGSMinimizer< ModelAdaptor< M >, QNUpdateType, Scalar, DimAtCompile > | protected |
| \_pk | stan::optimization::BFGSMinimizer< ModelAdaptor< M >, QNUpdateType, Scalar, DimAtCompile > | protected |
| \_pk\_1 | stan::optimization::BFGSMinimizer< ModelAdaptor< M >, QNUpdateType, Scalar, DimAtCompile > | protected |
| \_qn | stan::optimization::BFGSMinimizer< ModelAdaptor< M >, QNUpdateType, Scalar, DimAtCompile > | protected |
| \_xk | stan::optimization::BFGSMinimizer< ModelAdaptor< M >, QNUpdateType, Scalar, DimAtCompile > | protected |
| \_xk\_1 | stan::optimization::BFGSMinimizer< ModelAdaptor< M >, QNUpdateType, Scalar, DimAtCompile > | protected |
| alpha() const | stan::optimization::BFGSMinimizer< ModelAdaptor< M >, QNUpdateType, Scalar, DimAtCompile > | inline |
| alpha0() const | stan::optimization::BFGSMinimizer< ModelAdaptor< M >, QNUpdateType, Scalar, DimAtCompile > | inline |
| BFGSBase typedef | stan::optimization::BFGSLineSearch< M, QNUpdateType, Scalar, DimAtCompile > |  |
| BFGSLineSearch(M &model, const std::vector< double > &params\_r, const std::vector< int > &params\_i, std::ostream \*msgs=0) | stan::optimization::BFGSLineSearch< M, QNUpdateType, Scalar, DimAtCompile > | inline |
| BFGSMinimizer(ModelAdaptor< M > &f) | stan::optimization::BFGSMinimizer< ModelAdaptor< M >, QNUpdateType, Scalar, DimAtCompile > | inline |
| curr\_f() const | stan::optimization::BFGSMinimizer< ModelAdaptor< M >, QNUpdateType, Scalar, DimAtCompile > | inline |
| curr\_g() const | stan::optimization::BFGSMinimizer< ModelAdaptor< M >, QNUpdateType, Scalar, DimAtCompile > | inline |
| curr\_p() const | stan::optimization::BFGSMinimizer< ModelAdaptor< M >, QNUpdateType, Scalar, DimAtCompile > | inline |
| curr\_x() const | stan::optimization::BFGSMinimizer< ModelAdaptor< M >, QNUpdateType, Scalar, DimAtCompile > | inline |
| get\_code\_string(int retCode) | stan::optimization::BFGSMinimizer< ModelAdaptor< M >, QNUpdateType, Scalar, DimAtCompile > | inline |
| get\_qnupdate() | stan::optimization::BFGSMinimizer< ModelAdaptor< M >, QNUpdateType, Scalar, DimAtCompile > | inline |
| get\_qnupdate() const | stan::optimization::BFGSMinimizer< ModelAdaptor< M >, QNUpdateType, Scalar, DimAtCompile > | inline |
| grad(std::vector< double > &g) | stan::optimization::BFGSLineSearch< M, QNUpdateType, Scalar, DimAtCompile > | inline |
| grad\_evals() | stan::optimization::BFGSLineSearch< M, QNUpdateType, Scalar, DimAtCompile > | inline |
| grad\_norm() | stan::optimization::BFGSLineSearch< M, QNUpdateType, Scalar, DimAtCompile > | inline |
| HessianT typedef | stan::optimization::BFGSMinimizer< ModelAdaptor< M >, QNUpdateType, Scalar, DimAtCompile > |  |
| idx\_t typedef | stan::optimization::BFGSLineSearch< M, QNUpdateType, Scalar, DimAtCompile > |  |
| initialize(const std::vector< double > &params\_r) | stan::optimization::BFGSLineSearch< M, QNUpdateType, Scalar, DimAtCompile > | inline |
| BFGSMinimizer< ModelAdaptor< M >, QNUpdateType, Scalar, DimAtCompile >::initialize(const VectorT &x0) | stan::optimization::BFGSMinimizer< ModelAdaptor< M >, QNUpdateType, Scalar, DimAtCompile > | inline |
| iter\_num() const | stan::optimization::BFGSMinimizer< ModelAdaptor< M >, QNUpdateType, Scalar, DimAtCompile > | inline |
| logp() | stan::optimization::BFGSLineSearch< M, QNUpdateType, Scalar, DimAtCompile > | inline |
| minimize(VectorT &x0) | stan::optimization::BFGSMinimizer< ModelAdaptor< M >, QNUpdateType, Scalar, DimAtCompile > | inline |
| note() const | stan::optimization::BFGSMinimizer< ModelAdaptor< M >, QNUpdateType, Scalar, DimAtCompile > | inline |
| params\_r(std::vector< double > &x) | stan::optimization::BFGSLineSearch< M, QNUpdateType, Scalar, DimAtCompile > | inline |
| prev\_f() const | stan::optimization::BFGSMinimizer< ModelAdaptor< M >, QNUpdateType, Scalar, DimAtCompile > | inline |
| prev\_g() const | stan::optimization::BFGSMinimizer< ModelAdaptor< M >, QNUpdateType, Scalar, DimAtCompile > | inline |
| prev\_p() const | stan::optimization::BFGSMinimizer< ModelAdaptor< M >, QNUpdateType, Scalar, DimAtCompile > | inline |
| prev\_step\_size() const | stan::optimization::BFGSMinimizer< ModelAdaptor< M >, QNUpdateType, Scalar, DimAtCompile > | inline |
| prev\_x() const | stan::optimization::BFGSMinimizer< ModelAdaptor< M >, QNUpdateType, Scalar, DimAtCompile > | inline |
| rel\_grad\_norm() const | stan::optimization::BFGSMinimizer< ModelAdaptor< M >, QNUpdateType, Scalar, DimAtCompile > | inline |
| rel\_obj\_decrease() const | stan::optimization::BFGSMinimizer< ModelAdaptor< M >, QNUpdateType, Scalar, DimAtCompile > | inline |
| step() | stan::optimization::BFGSMinimizer< ModelAdaptor< M >, QNUpdateType, Scalar, DimAtCompile > | inline |
| vector\_t typedef | stan::optimization::BFGSLineSearch< M, QNUpdateType, Scalar, DimAtCompile > |  |
| VectorT typedef | stan::optimization::BFGSMinimizer< ModelAdaptor< M >, QNUpdateType, Scalar, DimAtCompile > |  |


---

[ Stan Home Page ]

*© 2011–2014,
Stan Development Team.*
